# REM sleep is associated with the volume of the cholinergic basal forebrain in aMCI individuals

**DOI:** 10.1186/s13195-023-01265-y

**Published:** 2023-09-08

**Authors:** Claire André, Marie-Ève Martineau-Dussault, Véronique Daneault, Hélène Blais, Sonia Frenette, Dominique Lorrain, Carol Hudon, Célyne Bastien, Dominique Petit, Alexandre Lafrenière, Cynthia Thompson, Jacques Montplaisir, Nadia Gosselin, Julie Carrier

**Affiliations:** 1 Center for Advanced Research in Sleep Medicine, Hôpital du Sacré-Coeur de Montréal, Recherche CIUSSS NIM, 5400 Boul Gouin O, Montréal, QC H4J 1C5 Canada; 2 Department of Psychology, Université de Montréal, Montreal, QC Canada; 3 Functional Neuroimaging Unit, University of Montreal Geriatric Institute, 4565 Queen-Mary Road, Montreal, QC H3W 1W5 Canada; 4 Research Centre On Aging, University Institute of Geriatrics of Sherbrooke, CIUSSS de L’Estrie-CHUS, Sherbrooke, QC Canada; 5 Department of Psychology, Université de Sherbrooke, Sherbrooke, QC Canada; 6 CERVO Research Centre, Québec City, QC Canada; 7 School of Psychology, Université Laval, Québec City, QC Canada; 8 Département de Psychiatrie, Université de Montréal, Montréal, QC Canada

**Keywords:** REM Sleep, Cholinergic Basal Forebrain, Nucleus Basalis of Meynert, Mild Cognitive Impairment, Neurodegeneration, Aging

## Abstract

**Background:**

Rapid-eye movement (REM) sleep highly depends on the activity of cholinergic basal forebrain (BF) neurons and is reduced in Alzheimer’s disease. Here, we investigated the associations between the volume of BF nuclei and REM sleep characteristics, and the impact of cognitive status on these links, in late middle-aged and older participants.

**Methods:**

Thirty-one cognitively healthy controls (66.8 ± 7.2 years old, 13 women) and 31 participants with amnestic Mild Cognitive Impairment (aMCI) (68.3 ± 8.8 years old, 7 women) were included in this cross-sectional study. All participants underwent polysomnography, a comprehensive neuropsychological assessment and Magnetic Resonance Imaging examination. REM sleep characteristics (i.e., percentage, latency and efficiency) were derived from polysomnographic recordings. T1-weighted images were preprocessed using CAT12 and the DARTEL algorithm, and we extracted the gray matter volume of BF regions of interest using a probabilistic atlas implemented in the JuBrain Anatomy Toolbox. Multiple linear regressions were performed between the volume of BF nuclei and REM sleep characteristics controlling for age, sex and total intracranial volume, in the whole cohort and in subgroups stratified by cognitive status.

**Results:**

In the whole sample, lower REM sleep percentage was significantly associated to lower nucleus basalis of Meynert (Ch4) volume (β = 0.32, *p* = 0.009). When stratifying the cohort according to cognitive status, lower REM sleep percentage was significantly associated to both lower Ch4 (β = 0.48, *p* = 0.012) and total BF volumes (β = 0.44, *p* = 0.014) in aMCI individuals, but not in cognitively unimpaired participants. No significant associations were observed between the volume of the BF and wake after sleep onset or non-REM sleep variables.

**Discussion:**

These results suggest that REM sleep disturbances may be an early manifestation of the degeneration of the BF cholinergic system before the onset of dementia, especially in participants with mild memory deficits.

**Supplementary Information:**

The online version contains supplementary material available at 10.1186/s13195-023-01265-y.

## Background

Cholinergic activity is the highest during rapid eye movement (REM) sleep, compared to non-REM sleep and wakefulness, while other neurotransmitter systems are almost silent [1–3]. Cortical cholinergic neurons mainly arise from the basal forebrain (BF) [4, 5], and widely project to the neocortex to drive the intense cortical activation characteristic of this sleep stage [3]. Silencing the cholinergic system with cholinergic antagonists, selective BF lesions, or optogenetic manipulation reduces or suppresses REM sleep, and induces slow wave activity [6–10]. Conversely, some studies have shown that stimulating BF cholinergic neurons with microinjections of neurotensin or photostimulation decreases slow wave sleep, favors transitions to REM sleep and enhances its duration [11, 12].

The BF undergoes severe and early degeneration in Alzheimer’s disease (AD), before medial temporal regions such as the entorhinal cortex or the hippocampus, and other neocortical areas [13–17]. Among all BF nuclei, the nucleus basalis of Meynert (Ch4) is affected particularly early [18], while neurons of the medial septum and the diagonal band of Broca (Ch1-2–3) are usually affected in later stages of the disease [18, 19]. Recent PET imaging studies using [^18^F]-FEOBV tracer have shown that AD patients exhibit a loss of cholinergic function [20, 21], which might be explained by Ch4 atrophy [20]. Similarly, the degeneration of the BF has been associated with cholinergic denervation, especially in medial temporal, frontal and temporo-parietal regions, in individuals with mild cognitive impairment (MCI) [22]. As cholinergic nuclei degenerate early in AD, REM sleep alterations are expected to arise in the early stages of the disease. This hypothesis is supported by results showing that REM sleep is altered in animal models of AD. Indeed, mice models of amyloid and tau pathologies exhibit a reduction of REM sleep duration and percentage, sometimes accompanied by increased REM sleep fragmentation, as compared to control mice [23–25]. Studies in humans report that patients with dementia present with sleep disturbances, including a reduction of REM sleep duration [26]. Previous studies suggest that this is also observed in the early stages of the disease, in individuals with amnestic MCI (aMCI) [27–30], especially those who will convert to dementia [28] or who carry the Apolipoprotein E ε4 allele [27]. Besides, epidemiological studies have shown that lower REM sleep duration predicts more severe cognitive decline over time [31], and that reduced REM sleep percentage is related to an increased risk of incident dementia [32] and mortality rate [33]. Yet, whether REM sleep quantity is associated with the volume of the BF in cognitively healthy middle-aged and older adults, and/or in adults with aMCI, needs to be investigated.

This cross-sectional study investigated whether REM sleep characteristics are associated with gray matter loss in BF nuclei in late middle-aged and older adults without moderate-to-severe obstructive sleep apnea (OSA) during REM sleep. Moreover, we characterized the impact of cognitive status on these links. We hypothesized that a reduction of REM sleep quantity, expressed as a percentage of total sleep time, would be associated with lower BF gray matter volume, especially in the Ch4 subregion. We expected these associations to be stronger in participants exhibiting objective memory deficits than in cognitively unimpaired individuals.

## Methods

### Study design

**Fig. 1:**
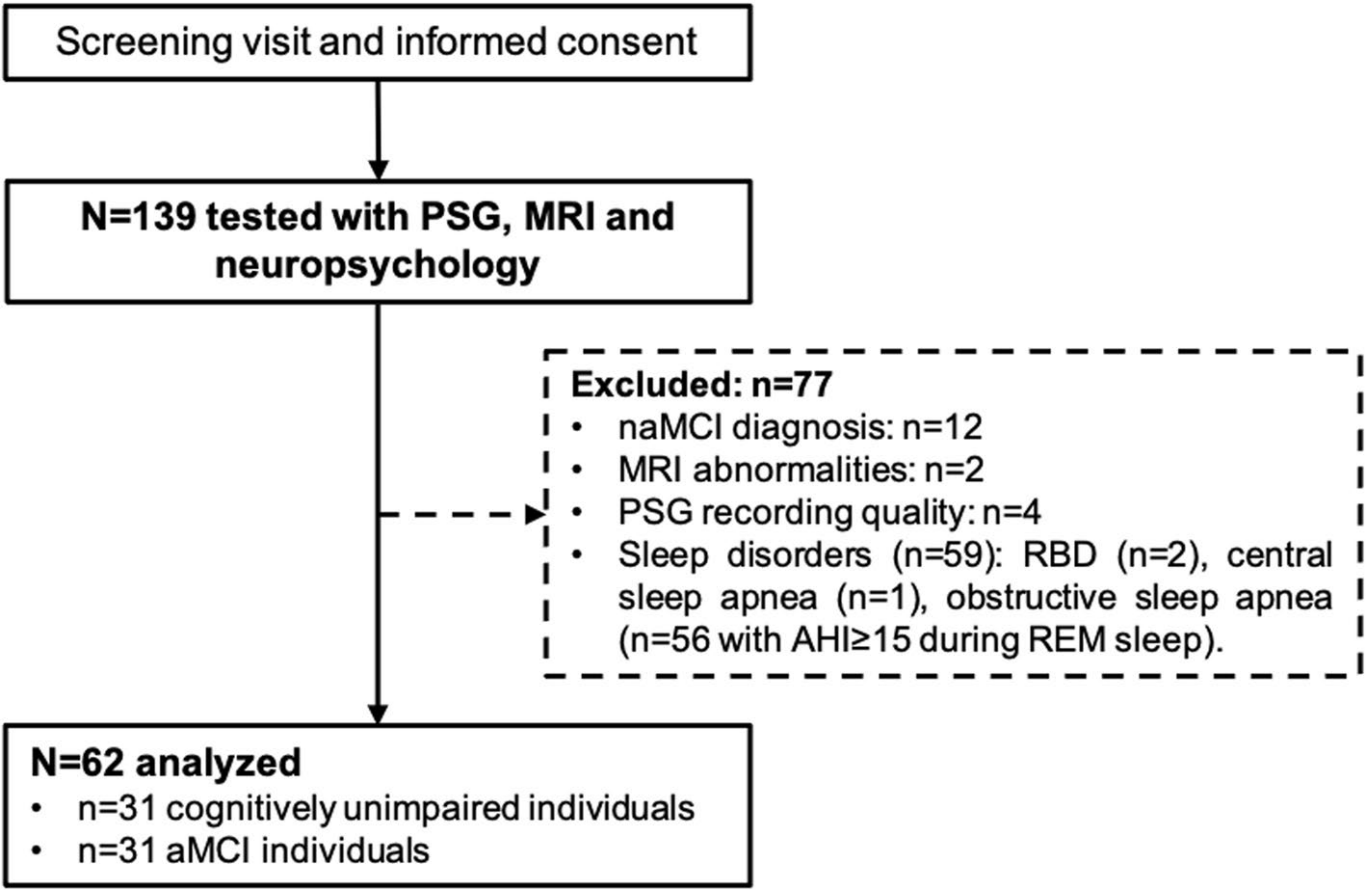
Flowchart of the study Abbreviations: AHI, apnea–hypopnea index; aMCI, amnestic mild cognitive impairment; MRI, magnetic resonance imaging; naMCI, non-amnestic mild cognitive impairment; PSG, polysomnography; RBD, REM sleep behavior disorder; REM, rapid eye movement

One hundred and eighteen participants aged over 55 years old, with at least 7 years of education, fluent in French or English, and with a preserved autonomy in daily life were recruited in the context of four protocols between 2012 and 2020 [34]. All protocols were approved by institutional ethics committees (#2012–697, #12–13-008, #2010–468 and #MP-32–2018-1537), and written informed consent was obtained from each participant prior to examinations, according to the declaration of Helsinki. Specifically, between 2012 and 2016, 73 participants were recruited in Montreal as part of three protocols on aging and MCI. The remaining 45 participants were recruited from three sites as part of a multicentric project, between 2018 and 2020 (*n* = 23 in Montreal, *n* = 16 in Sherbrooke and *n* = 6 in Quebec City). All participants were recruited through local memory and sleep clinics, previous research protocols or newspaper advertisements. Exclusion criteria were the presence or history of neurological (e.g., dementia, epilepsy, traumatic brain injury or encephalopathy), psychiatric disorders (e.g., diagnosed major depression or anxiety), sleep disorders diagnosis or confirmed by the PSG (e.g., insomnia, periodic limb movements disorder, restless legs syndrome, REM sleep behaviour disorder), restless legs syndrome, cerebrovascular or pulmonary diseases (e.g., history of stroke, chronic obstructive pulmonary disease), uncontrolled diabetes or hypertension, body mass index greater than 40 kg/m^2^, drug or alcohol abuse, heavy consumption of caffeinated beverages, use of psychotropic medications affecting sleep, cognition or brain functioning (e.g., antidepressants, hypnotics, opioids), presenting with a contraindication for MRI scanning (e.g., claustrophobia, metallic implants), and brain abnormalities detected on MRI images. Participants underwent a phone screening followed by an in-person interview, neuropsychological evaluation, in-laboratory polysomnographic recording and structural MRI scanning. Importantly, as obstructive sleep apnea (OSA) is particularly frequent during REM sleep [35, 36] and may interfere with the integrity of the cholinergic BF [37], 56 participants with an apnea–hypopnea index ≥ 15 during REM sleep were excluded before statistical analyses. The final sample included 62 participants: 41 participants were recruited in Montreal between 2012 and 2016 as part of the three protocols on aging and MCI, and 21 participants were recruited as part of the multicentric project between 2018 and 2020 (*n* = 10 in Montreal, *n* = 9 in Sherbrooke and *n* = 2 in Quebec City) (see the Flowchart in Fig. 1).

### Neuropsychological evaluation

Participants underwent a comprehensive neuropsychological evaluation, encompassing global cognitive functioning, attention and processing speed, executive functioning, learning and memory, language, and visuo-spatial abilities. In each cognitive domain, core neuropsychological tests were available for all participants, while some additional tests varied across the 4 protocols (see Supplementary Table 1 for details). A clinical cognitive diagnosis was established by consensus between senior neuropsychologists, based on neuropsychological performance and using as many scores as possible for each cognitive domain. Briefly, a cognitive domain was considered altered when participants presented with two or more Z-scores ≤ 1.5 standard deviation (SD) in a given cognitive domain, or if participants presented a MoCA score < 26 accompanied by one Z-score ≤ 1.5 SD in at least two cognitive domains including memory. Participants for whom all cognitive domains were preserved were classified as cognitively unimpaired (*n* = 31). Participants with at least one impaired cognitive domain that included memory were classified as aMCI participants (*n* = 31).

### Questionnaires

Participants filled out different questionnaires to characterise the sample. They included the Epworth Sleepiness Scale [38], assessing excessive daytime sleepiness and questionnaires measuring anxiety and depression levels. As they varied between protocols, we dichotomized these variables based on validated cut-offs for the presence of mild symptoms. We defined the presence of significant anxiety symptoms using cut-offs of ≥ 8 on the Beck Anxiety Inventory [39] or the Geriatric Anxiety Inventory [40, 41]. Similarly, we defined the presence of significant depressive symptoms using cut-offs of ≥ 14 on the Beck Depression Inventory II [42] and of ≥ 5 on the Geriatric Depression Scale [43].

### Polysomnographic recording

All participants underwent an in-laboratory polysomnographic recording with a minimum of 12 EEG electrodes common to all cohorts (F3, F4, C3, C4, T3, T4, T5, T6, P3, P4, O1, O2) referenced to the mastoids and placed on the scalp according to the international 10–20 system. Before 2019, 43 participants underwent a polysomnographic recording using a Grass system (bandpass 0.3–100 Hz), and signals were digitized at a sampling rate of 256 Hz using the Harmonie software (Stellate Systems, Montreal, Quebec, Canada). Starting from 2019, 19 participants underwent a polysomnographic recording using a Natus system (Brain Monitor, Trex and Embla NDx; bandpass 0.3–200 Hz, digitized at a sampling rate of 512 Hz). For all participants, the recording also included an electrooculogram, electrocardiogram, and chin and leg electromyograms. We recorded respiration and oxygen saturation using oronasal canula and thermistors, thoraco-abdominal belts and a finger pulse oximeter.

Sleep stages and respiratory events were visually scored in 30-s epochs by certified medical electrophysiology technologists at the Montreal site, according to standard international criteria of the American Academy of Sleep Medicine [44]. Several variables reflecting REM sleep integrity were computed, including REM sleep duration (encompassing all epochs scores as REM sleep, expressed in minutes and as a percentage of total sleep time), latency and efficiency. The efficiency of each REM sleep period was calculated as follows: (number of minutes scored as REM sleep / duration of REM periods in minutes) * 100. REM sleep periods were defined as starting at the first REM epoch, had a minimum duration of 5 min (except the first and last REM sleep period, which have no minimum duration), and had to be separated by at least 15 min of NREM sleep [45]. For each participant, REM sleep efficiency was computed as the mean efficiency of all REM sleep periods of the recording.

Sleep apneas were defined as drops of ≥ 90% of airflow for a minimum of 10 s [44]. Hypopneas were defined as a ≥ 30% reduction of airflow for a minimum of 10 s, followed by either a cortical arousal or a ≥ 3% oxygen desaturation [44]. Obstructive sleep apnea severity was estimated by the apnea–hypopnea index, corresponding to the number of apneas and hypopneas per hour of sleep. The apnea–hypopnea index was computed for both total sleep time and REM sleep specifically. All 62 participants included in the analyses presented with an apnea–hypopnea index < 15 during REM sleep.

### Structural MRI

#### Acquisition

T1-weighted images were acquired between December 2012 and December 2019 in three Canadian sites (Montreal, Quebec City, and Sherbrooke). Between 2012 and 2016, 41 participants were scanned at the Functional Neuroimaging Unit of the Montreal Geriatric University Institute with a 3 T Siemens Magnetom Trio Tim scanner (Siemens Healthcare, USA), as part of a project on obstructive sleep apnea and MCI. A 3D T1-weighted MP-RAGE sequence was acquired with a 32-channel head coil using the parameters of the Massachusetts General Hospital (Boston, Massachusetts, USA): repetition time = 2530 ms; root mean square of four echo times = 1.64 ms, 3.50 ms, 5.36 ms, 7.22 ms; inversion time = 1200 ms; matrix size = 256 × 256; field of view = 256 × 256 mm; voxel size = 1.0 mm isotropic; flip angle = 7°; and 176 sagittal orientations.

Between August and December 2019, a multicenter study was conducted on sleep and MCI. Neuroimaging acquisitions were performed following the Canadian Dementia Imaging Protocol (www.cdip-pcid.ca) [46], which was established by the Canadian Consortium on Neurodegeneration in Aging (CCNA) to harmonize acquisitions and minimize differences in the context of multicentric studies. In Montreal, 10 participants were scanned with the upgraded 3 T Siemens Prisma Fit scanner. A 3D T1-weighted sequence was acquired with the following parameters: repetition time = 2300 ms; echo time = 2.98 ms; inversion time = 900 ms; matrix size = 256 × 256; field of view = 256 × 256 mm; voxel size = 1.0 mm isotropic; flip angle = 8°; and 192 sagittal orientations. In Quebec City, 2 participants were scanned at the CERVO Brain Research Centre using a 3 T Philips Achieva dStream scanner. A 3D T1-weighted sequence was acquired using the following parameters: repetition time = 7.3 ms; echo time = 3.3 ms; inversion time = 945 ms; matrix size = 256 × 256; field of view = 256 × 256 mm; voxel size = 1.0 mm isotropic; flip angle = 9°; and 180 sagittal orientations. In Sherbrooke, 9 participants were scanned at the University Institute of Geriatrics of Sherbrooke with a 3 T Ingenia Philips scanner. The MP-RAGE sequence was acquired using the following parameters: repetition time = 7.1 ms; echo times = 3.2 ms; matrix size = 256 × 256; field of view = 256 × 256 mm; voxel size = 1.0 mm isotropic; flip angle = 9°; 192 sagittal orientations.

#### Preprocessing

T1-weighted images were preprocessed using the Computational Anatomy Toolbox version 12.7 (CAT12, Jena University Hospital, Germany; release 1653; www.neuro.uni-jena.de/cat/index.html) for SPM12 (Wellcome Trust Centre for Neuroimaging, London, UK; release 6906; https://www.fil.ion.ucl.ac.uk/spm), under MATLAB R2018b (MathWorks, Natick, MA, USA; https://www.mathworks.com). Images were segmented into gray matter, white matter, and CSF, and a bias correction of intensity nonuniformities was applied. Spatial registration to a reference template within the Montreal Neurological Institute (MNI) space was computed using the diffeomorphic anatomical registration through exponentiated lie algebra algorithm (DARTEL) [47]. This step created an average brain template from all study participants before normalization in the MNI space. Gray matter maps were then modulated (i.e., scaled by the volume changes due to spatial registration), and a visual inspection was performed on all resulting maps for quality check. The total intracranial volume (TIV) was calculated using the implemented TIV estimation module in CAT12.

#### Basal forebrain volumetric measures

BF regions of interest (ROIs) were obtained using a probabilistic atlas created based on postmortem data [48], implemented in the JuBrain Anatomy toolbox (version 2.2) [49]. According to Mesulam’s nomenclature [4], three masks of distinct BF ROIs were used: the left and right Ch4 ROI, corresponding to the nucleus basalis of Meynert, and bilateral Ch1-2–3 ROI, corresponding to the medial septum, vertical and horizontal limbs of the diagonal band of Broca. Masks were co-registered and resliced on the DARTEL template using SPM12 before gray matter extraction. Estimates of gray matter volume (in mm^3^) were extracted from Ch1-2–3, Ch4 and the total BF masks on unsmoothed modulated gray matter maps using the “get_totals” script (http://www0.cs.ucl.ac.uk/staff/g.ridgway/vbm/get_totals.m).

### Statistical analyses

The normality of each variable was tested using the Shapiro–Wilk test and all non-normal variables were log-transformed before analysis (i.e., Ch4 volume, REM sleep latency, wake after sleep onset duration, N1- and N3-sleep duration). Differences between cognitively unimpaired and aMCI participants on demographics, sleep, cognitive, and imaging variables were assessed using Student t-tests for continuous variables and chi-square tests for categorical variables.

Multiple linear regressions were performed with REM sleep variables (i.e., REM sleep percentage, efficiency and latency) as predictors, and the volume of BF subregions (i.e., the total BF, Ch1-2–3 and Ch4) as outcomes, controlling for age, sex and the TIV. Separate models were carried out for each REM sleep variable and BF subregion in the whole sample and in subgroups stratified by cognitive status. Of note, adding the type of scan as a covariate did not change the results (data not shown), so this covariate was not included in the final regression models to avoid overfitting.

To investigate the specificity of the associations between REM sleep and BF volume, multiple regression analyses were also performed between (i) BF volumes and the percentage of other sleep stages (i.e., N1, N2 and N3 sleep) and wake after sleep onset, and (ii) REM sleep percentage and the volume of other brain regions vulnerable to AD or control areas (i.e., anterior cingulate, amygdala, cuneus, hippocampus, inferior temporal, posterior cingulate, precuneus, superior frontal and supramarginal gyri volumes, as well as total gray matter volume).

For transparency, we report the results at a *p* < 0.05 uncorrected threshold, but only those surviving a False Discovery Rate (FDR) adjustment were considered significant and robust. Statistical analyses were performed using JASP (version 0.16.1) and R (version 1.3.1056).

## Results

### Participants

The flowchart of the study is described in Fig. 1, and participants’ characteristics are displayed in Table 1. The mean age of the sample was 67.6 ± 8.1 years, including 20 women (32.3% of the sample). Cognitively unimpaired and aMCI individuals did not differ in terms of age, sex, body-mass index, anxiety and depressive symptoms, sleep architecture, TIV and BF volume. However, as expected, aMCI patients had significantly lower MoCA scores and episodic memory performance.

**Table 1:** Participants’ characteristics

	**Full sample (*n* = 62)**	**Cognitively unimpaired (*n* = 31)**	**aMCI (*n* = 31)**	**T or χ**^**2**^	**P**
**Demographics and cognition**					
Age: years	67.6 ± 8.1	66.8 ± 7.2	68.3 ± 8.8	-0.74	*p* = 0.46
Sex: number (%) of women	20 (32.3)	13 (41.9)	7 (22.6)	2.66	*p* = 0.10
Education: years	14.5 ± 3.5	15.3 ± 2.9	13.8 ± 4	1.71	*p* = 0.09
Body mass index: kg/m^2^	25.2 ± 3.4	25.2 ± 3.7	25.1 ± 3.1	0.08	*p* = 0.94
Anxiety symptoms: % with^a^	9 (14.5)	4 (12.9)	5 (16.1)	0.13	*p* = 0.72
Depressive symptoms: % with^a^	3 (4.8)	0 (0)	3 (9.7)	3.15	*p* = 0.08
MoCA: score ^b^	26.5 ± 2.7	27.8 ± 1.8	25.1 ± 2.8	4.49	***p* < 0.001**
RAVLT: Sum of the 5 free recalls	44.9 ± 12.1	53.2 ± 8.0	36.6 ± 9.5	7.40	***p* < 0.001**
RAVLT: immediate free recall	8.9 ± 3.9	11.5 ± 2.4	6.4 ± 3.4	6.84	***p* < 0.001**
RAVLT: delayed free recall^a^	8.5 ± 3.9	11.0 ± 2.6	5.8 ± 3.2	6.98	***p* < 0.001**
**Sleep**					
Total Sleep Time: min	363.8 ± 63.1	374.1 ± 62.6	353.4 ± 63.0	1.30	*p* = 0.20
Sleep efficiency: %	79.5 ± 11.1	80.7 ± 11.0	78.2 ± 11.3	0.88	*p* = 0.38
Total sleep AHI: Nb/h	7.8 ± 9.1	7.1 ± 7.6	8.5 ± 10.4	-0.59	*p* = 0.56
REM sleep AHI: Nb/h	5.0 ± 4.5	5.5 ± 4.7	4.6 ± 4.3	0.75	*p* = 0.46
NREM-1: min	59.7 ± 32.6	53.9 ± 26.8	65.5 ± 37.1	-1.41	*p* = 0.16
NREM-1: % TST	16.6 ± 8.7	14.4 ± 6.6	18.7 ± 10.0	-1.97	*p* = 0.054
NREM-2: min	212.1 ± 54.3	222.2 ± 49.7	202.1 ± 57.7	1.47	*p* = 0.15
NREM-2: % TST	58.0 ± 9.0	59.2 ± 8.0	56.7 ± 9.8	1.12	*p* = 0.27
NREM-3: min	31.9 ± 30.3	36.5 ± 35.0	27.2 ± 24.4	1.21	*p* = 0.23
NREM-3: % TST	9.1 ± 8.8	10.3 ± 9.7	8.0 ± 7.7	1.00	*p* = 0.32
REM sleep: min	60.1 ± 22.9	61.6 ± 24.5	58.7 ± 21.5	0.50	*p* = 0.62
REM sleep: % TST	16.3 ± 5.1	16.1 ± 5.1	16.6 ± 5.1	-0.40	*p* = 0.69
REM sleep latency: min	135.1 ± 86.5	117.9 ± 74.6	152.4 ± 95.1	-1.59	*p* = 0.12
REM sleep efficiency: %	81.0 ± 12.0	80.2 ± 11.7	81.8 ± 12.5	-0.53	*p* = 0.60
**Neuroimaging**					
Total Intracranial volume: cm^3^	1472 ± 122.8	1470.1 ± 136.0	1474.0 ± 110.3	-0.12	*p* = 0.90
Total BF: mm^3^	417.1 ± 43.9	425.2 ± 36.1	409.0 ± 49.8	1.47	*p* = 0.15
Bilateral Ch1-2–3 nuclei: mm^3^	152.6 ± 17.4	155.1 ± 16.1	150.2 ± 18.5	1.14	*p* = 0.26
Bilateral Ch4 nuclei: mm^3^	264.5 ± 32.8	270.1 ± 25.2	258.9 ± 38.6	1.35	*p* = 0.12

Data are expressed as mean ± standard deviation, unless otherwise specified. All non-normal variables were log-transformed before statistical analyses, and between-group differences were assessed using Student t-tests for continuous variables and chi-square tests for categorical variables. Results in bold indicate significant differences at the *p* < 0.05 uncorrected level

*Abbreviations*: *AHI* apnea–hypopnea index, *aMCI* amnestic mild cognitive impairment, *BF* basal forebrain; *h* hours, *MoCA* Montreal Cognitive Assessment; *nb* number, *NREM* non-rapid eye movement, *RAVLT* Rey Auditory Verbal Learning Test, *REM* rapid eye movement

^a^Missing data for 1 aMCI participant

^b^Missing data for 3 participants (1 cognitively unimpaired participant and 2 aMCI)

#### Basal forebrain volume and REM sleep characteristics

**Fig. 2:**
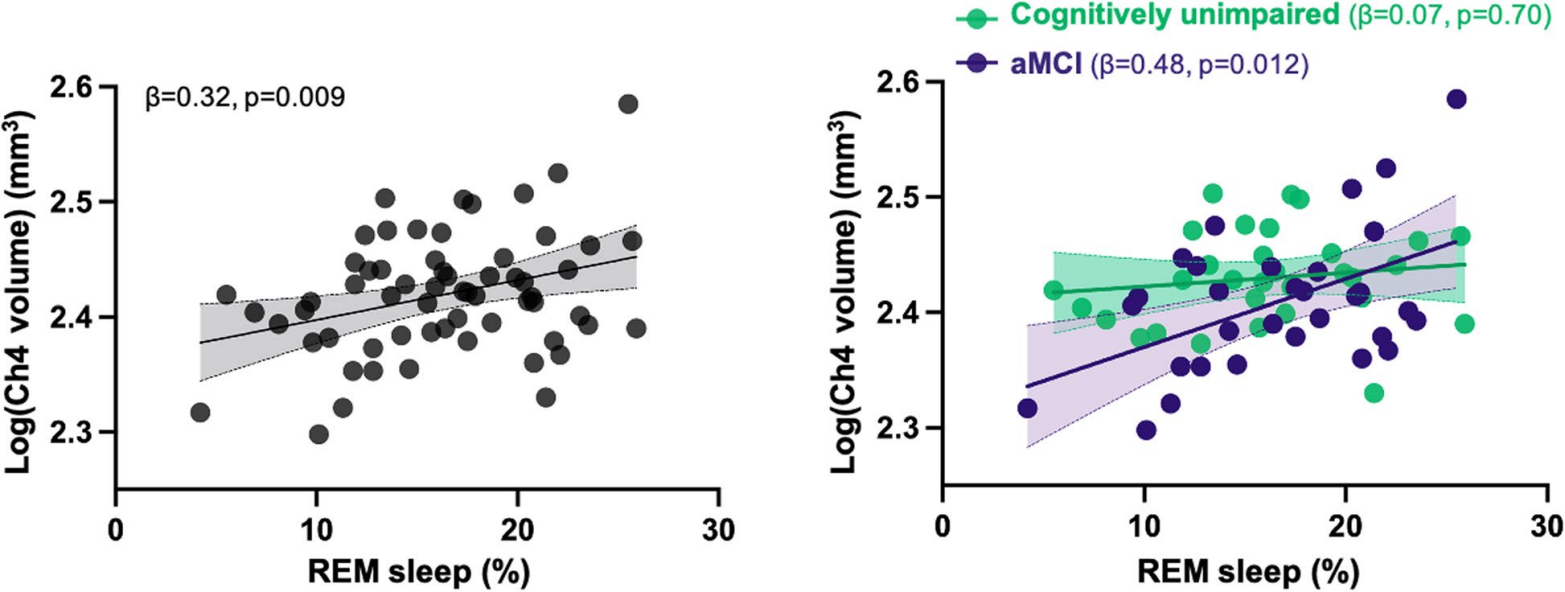
Associations between REM sleep percentage and Ch4 volume Scatterplots illustrating the associations between log-transformed Ch4 volume (in mm^3^) and REM sleep percentage in the whole cohort (left) and according to cognitive status (right). Abbreviations: aMCI, amnestic mild cognitive impairment; REM, rapid eye movement

We first assessed whether REM sleep characteristics were associated with the volume of BF subregions. Lower REM sleep percentage was positively associated to lower gray matter volume in the nucleus basalis of Meynert (Ch4) (β = 0.32, *p* = 0.009), such that lower Ch4 volume was related to lower REM sleep percentage (Table 2 and Fig. 2). In addition, lower REM sleep percentage was marginally associated with lower total BF volume (β = 0.27, *p* = 0.02), but this association did not survive an FDR correction for multiple comparisons (Table 2). No significant association was observed between BF volume and REM sleep latency or efficiency.

**Table 2:** Multiple linear regressions between REM sleep characteristics and basal forebrain volume

Dependent variable	Independent variables	Unstandardized coefficient (95% CI)	Standard Error	Standardized coefficient	P_unc_	P_FDR-corrected_
Ch1-2–3 volume	REM-S percentage	0.2 (-0.55 – 0.95)	0.38	0.06	0.59	
	REM-S latency	-4.14 (-19.4 – 11.13)	7.62	-0.06	0.59	
	REM-S efficiency	-0.04 (-0.35 – 0.28)	0.16	-0.03	0.82	
**Ch4 volume**	**REM-S percentage**	**0.003 (8.9e**^**−4**^** – 0.006)**	**0.001**	**0.32**	**0.009**	**0.03**
	REM-S latency	-0.04 (-0.09 – 0.01)	0.03	-0.19	0.13	
	REM-S efficiency	2.6e^−4^ (-8.4e^−4^ – 0.001)	5.5e^−4^	0.06	0.64	
Total BF volume	REM-S percentage	2.33 (0.38 – 4.28)	0.97	0.27	0.02	0.06
	REM-S latency	-29.03 (-69.89 – 11.82)	20.4	-0.16	0.16	
	REM-S efficiency	0.09 (-0.76 – 0.94)	0.43	0.02	0.84	

Results of multiple regressions performed with REM sleep variables (i.e., REM sleep percentage, latency and efficiency) as predictors, and the volume of BF subregions (i.e., Ch1-2–3, Ch4 and the total BF) as outcomes, controlling for age, sex and the total intracranial volume. Results indicated in bold survive an FDR correction for multiple comparisons

*Abbreviations*: *BF* basal forebrain, *CI* confidence interval, *FDR* false discovery rate, *REM-S* rapid eye movement sleep

#### Basal forebrain volume and REM sleep characteristics in subgroups stratified by cognitive status.

Analyses were then performed separately with cognitively unimpaired and aMCI individuals, and we found that REM sleep percentage was positively associated with bilateral Ch4 volume (β = 0.48, *p* = 0.01) and total BF volume (β = 0.44, *p* = 0.01) in individuals with aMCI, but not in cognitively unimpaired participants **(**Table 3 and Fig. 2).

**Table 3:** Multiple linear regressions between REM sleep characteristics and basal forebrain volume in subsamples stratified by cognitive status

Dependent Variable	Independent variables	Unstandardized coefficient (95% CI)	Standard Error	Standardized coefficient	P_unc_	P_FDR-corrected_
**Cognitively unimpaired participants**						
Ch1-2–3 volume	REM-S percentage	-0.19 (-1.13 – 0.75)	0.46	-0.06	0.68	
	REM-S latency	-16.68 (-37.39 – 4.04)	10.08	-0.21	0.11	
	REM-S efficiency	-0.31 (-0.7 – 0.09)	0.19	-0.22	0.12	
Ch4 volume	REM-S percentage	5.4e^−4^ (-0.002 – 0.003)	0.001	0.07	0.70	
	REM-S latency	-0.06 (-0.12 – 0.005)	0.03	-0.28	0.07	
	REM-S efficiency	-9.3e^−5^ (-0.001 – 0.001)	6.2e^−4^	-0.03	0.88	
Total BF volume	REM-S percentage	0.19 (-2.05 – 2.43)	1.09	0.03	0.86	
	REM-S latency	-52.17 (-99.75 – -4.59)	23.15	-0.29	0.03	0.09
	REM-S efficiency	-0.38 (-1.36 – 0.6)	0.48	-0.12	0.43	
**aMCI participants**						
Ch1-2–3 volume	REM-S percentage	0.68 (-0.63 – 1.99)	0.64	0.19	0.3	
	REM-S latency	1.69 (-20.62 – 24)	10.85	0.03	0.88	
	REM-S efficiency	0.15 (-0.38 – 0.69)	0.26	0.1	0.56	
**Ch4 volume**	**REM-S percentage**	**0.006 (0.001** – **0.01)**	**0.002**	**0.48**	**0.012**	**0.036**
	REM-S latency	-0.02 (-0.11 – 0.06)	0.04	-0.11	0.55	
	REM-S efficiency	7.5e^−5^ (-0.001 – 0.003)	9.5e^−4^	0.15	0.44	
**Total BF volume**	**REM-S percentage**	**4.31 (0.93** – **7.68)**	**1.64**	**0.44**	**0.014**	**0.042**
	REM-S latency	-14.4 (-77.61 – 48.81)	30.75	-0.08	0.64	
	REM-S efficiency	0.54 (-0.99 – 2.06)	0.74	0.14	0.48	

Results of multiple regressions performed separately within each group, with REM sleep variables (i.e., REM sleep percentage, latency and efficiency) as predictors, and the volume of BF subregions (i.e., Ch1-2–3, Ch4 and the total BF) as outcomes, controlling for age, sex and the total intracranial volume. Results indicated in bold survive an FDR correction for multiple comparisons

*Abbreviations*: *aMCI* amnestic Mild Cognitive Impairment, *BF* basal forebrain, *CI* confidence interval, *FDR* false discovery rate, *REM* rapid eye movement

#### Sensitivity and specificity analyses

We then investigated the robustness and specificity of the association between REM sleep percentage and Ch4 volume.

First, we observed that REM sleep duration, expressed in minutes rather than as a percentage of total sleep time, was similarly positively associated with Ch4 (full sample: β = 0.30, *p* = 0.017; aMCI individuals: β = 0.45, *p* = 0.019) and total BF volume (full sample: β = 0.28, *p* = 0.016; aMCI individuals: β = 0.44, *p* = 0.015) in the whole cohort and in aMCI individuals (Supplementary Table 2).

Second, we checked whether NREM or wakefulness variables were associated with the volume of the BF. No significant association was found between the volume of BF subregions and NREM sleep variables or the amount of wake after sleep onset in the whole cohort (Supplementary Table 3).

Third, we verified whether REM sleep percentage reduction was significantly associated with the volume of other brain regions, and found no significant associations (Supplementary Table 4).

Finally, we checked whether lower REM sleep duration was associated with an increase in other sleep/wake stages. We observed that lower REM sleep duration was robustly associated with greater N3 sleep duration (Supplementary Table 5). More marginal associations (i.e., only with REM sleep duration expressed in minutes but not as a percentage of total sleep time) were found between lower REM sleep duration and increased wake and N2 sleep duration.

## Discussion

Our results show that reduced REM sleep percentage is related to lower nucleus basalis of Meynert (Ch4) volume, especially in aMCI individuals but not in cognitively unimpaired participants. In aMCI individuals, we also observed an association between lower REM sleep percentage and lower total BF volume. Importantly, these links were specific to REM sleep, as no significant associations between BF volume and non-REM sleep architecture or the amount of wake after sleep onset were observed.

Sleep disturbances are increasingly recognized as both a consequence and risk factor for cognitive decline and AD [50]. However, slow wave sleep has received the most attention to date [51]. The present study suggests that REM sleep changes may be intimately associated with the degeneration of BF cholinergic nuclei. The cholinergic system is known to play a key role in REM sleep physiology [2], notably by underlying its characteristic intense and fast cortical activity [3]. Furthermore, cholinergic neurons are active during REM sleep but are almost silent during NREM sleep [52]. Consistently, the present study shows that the volume of the BF is specifically associated with REM sleep, as no significant association was observed with non-REM sleep architecture. Therefore, our results confirm that REM sleep alterations could represent an early marker of the volume of the BF cholinergic system.

Furthermore, the volume of the Ch4 nuclei (i.e., the nucleus basalis of Meynert) was specifically and robustly associated with REM sleep. Indeed, we did not observe significant associations between REM sleep and the volume of Ch1-2–3 nuclei. Neurons of the nucleus basalis of Meynert are known to widely innervate neocortical areas [4], and are thus likely underlying the intense cortical activation observed in REM sleep. However, some studies suggest that these neurons start to degenerate early in patients with MCI or Subjective Cognitive Decline [53], while Ch1-2–3 neurons are usually affected in later stages of the disease [18, 19]. Ch4 neurons are especially sensitive to tau pathology [16], probably due to their large axons exhibiting high metabolic rates [54, 55]. They are also affected before cortical regions, including the entorhinal cortex [14, 15]. In addition, the longitudinal degeneration of BF subregions has been shown to covary with cortico-amygdalar degeneration patterns, reflecting the spatial organization of cholinergic BF projections and [^18^F]FEOBV-PET indices of cholinergic denervation in participants with MCI [22].

In our sample, we did not observe significant associations between REM sleep percentage and the volume of medial temporal areas (i.e., the hippocampus and amygdala) or other cortical regions such as the anterior and posterior cingulate cortex, cuneus, precuneus, inferior temporal, superior frontal and supramarginal gyri, as well as total gray matter volume. This suggests that REM sleep percentage was not influenced by neocortical volume. Importantly, the associations between REM sleep and the volume of subcortical nuclei are certainly not restricted to cholinergic BF neurons in older adults, and may be influenced by other subcortical areas. Although still not fully understood, the mechanisms regulating REM sleep are known to involve complex reciprocal interactions between several neurotransmitter systems, with core nuclei located in the brainstem, BF and hypothalamus [56]. An extensive literature also highlights the crucial role of pontine nuclei in the brainstem for the generation and maintenance of REM sleep [57–59]. The BF receives crucial input from multiple brainstem nuclei, among other regions (e.g., the hypothalamus and amygdala), and in turn sends widespread cholinergic projections to the neocortex sustaining cortical activation, which may participate to REM sleep maintenance [4, 5]. Importantly, several brainstem nuclei involved in the regulation of the sleep–wake cycle also accumulate tau pathology and degenerate in AD before cortical regions [60, 61]. Because of MRI resolution limitations, we were not able to reliably assess the volume of brainstem nuclei in the present study. However, due to the redundant nature of REM sleep regulating circuits, the dysfunction of brainstem nuclei may, at least in part, influence the BF-related reduction of REM sleep percentage we observe in our sample, and should be further investigated.

Stratifying the sample according to cognitive status has revealed that despite similar BF and REM sleep volume between aMCI and controls, the association between REM sleep quantity and Ch4 volume was only significant in aMCI patients. One possible explanation is that cognitively unimpaired participants with BF volume loss may be able to compensate by upregulating choline acetyltransferase enzyme activity, which is critical for the synthesis of acetylcholine released into the synapse [62], preventing measurable REM sleep changes. Indeed, it has been previously shown that in predementia stages of AD, when BF neurons are injured, enzyme synthesis in the remaining cholinergic neurons can be upregulated to compensate for neuronal loss [63]. However, it is still possible that in cognitively unimpaired individuals, BF alterations may be associated with more subtle changes in REM sleep microstructure, rather than macrostructure. It is worth mentioning that contrary to some previous reports in the literature [13, 18, 64], we did not find a significant reduction of BF volumes in aMCI participants compared to controls in our sample. Some possible reasons explaining this absence of difference may include the lack of characterization of biomarker status of the participants, limited sample size, the cross-sectional nature of the investigation (with longitudinal designs identifying MCI individuals converting to dementia being more powerful), and stringent OSA screening. Indeed, recent animal studies show that BF atrophy is associated with OSA [37]. We have applied stringent exclusion criteria by removing from our analysis sample participants with moderate-to-severe levels of OSA during REM sleep, as this is a major confounder of REM sleep integrity measures. As a consequence, this may have, to some extent, impacted the variability of BF volumes in our cohort.

### Strengths and limitations

The strengths of the present study are the combination of polysomnography and structural MRI, and the stratification of participants according to cognitive status, in a sample carefully screened for sleep disorders and other health conditions. Some limitations should however be mentioned. First, we did not assess the levels of amyloid and tau pathologies, such that we could not confirm that all aMCI participants are biologically engaged in the AD continuum. Further studies should assess the associations between the integrity of REM sleep and the BF cholinergic system in older individuals stratified by AD biomarker status. Second, our study design was cross-sectional, preventing from determining causal relationships between REM sleep and BF structural alterations. Third, this study is multicentric and participants were scanned using different scanners. However, we did follow an imaging protocol specifically designed to limit the impact of using diverse scanners [46], and adding the scan as a covariate did not change the results. Fourth, although sex was added as a covariate in all analyses and that the proportion of women did not significantly differ between aMCI and healthy controls, we acknowledge that the proportion of women was relatively low in our cohort (i.e., around 32.3% of the whole sample). Notably, the low proportion of women, especially in the aMCI group (around 23% in the aMCI group, compared to approximately 42% in healthy controls), may have impacted our results and prevented from examining the moderating effect of sex. Therefore, future studies performed in larger samples should further examine the differential impact of sex on the associations between sleep and neurodegeneration. Lastly, this study focused on studying sleep architecture, and future studies will need to assess the relationships between BF volume and other REM sleep variables (e.g., spectral power or the density of rapid eye movements).

## Conclusions

Overall, our results suggest that the degeneration of the BF cholinergic system in prodromal AD is associated with early REM sleep disturbances, before the onset of dementia. Reduced REM sleep duration may represent an early marker of the degeneration of the nucleus basalis of Meynert in older individuals.

### Supplementary Information

**Additional file 1: Supplementary Table 1.** Neuropsychological tests used for cognitive diagnosis. **Supplementary Table 2.** Multiple linear regressions between REM sleep duration and BF volume. **Supplementary Table 3.** Multiple linear regressions between NREM sleep and wakefulness variables and BF volume. **Supplementary Table 4.** Partial correlations between REM sleep duration and the volume of control brain regions.

## Data Availability

Data used in the present study will be available from the corresponding author upon request.

## Abbreviations

AD: Alzheimer’s disease
BF: Basal forebrain
FDR: False Discovery Rate
MCI: Mild cognitive impairment
NREM: Non rapid eye movement
REM: Rapid eye movement
ROI: Region of interest
SD: Standard deviation
TIV: Total intracranial volume
