# Supplementary material for: REM sleep is associated with the volume of the cholinergic basal forebrain in aMCI individuals
Source: Alzheimers Res Ther. 2023 Sep 8;15:151. doi: 10.1186/s13195-023-01265-y (PMC10485959; doi:10.1186/s13195-023-01265-y)
Supplement: Supplementary file 1 — Additional file 1: Supplementary Table 1. Neuropsychological tests used for cognitive diagnosis. Supplementary Table 2. Multiple linear regressions between REM sleep duration and BF volume. Supplementary Table 3. Multiple linear regressions between NREM sleep and wakefulness variables and BF volume. Supplementary Table 4. Partial correlations between REM sleep duration and the volume of control brain regions. [file 13195_2023_1265_MOESM1_ESM.docx]

**Supplementary Material**

**Supplementary Table 1: Neuropsychological tests used for cognitive diagnosis.**

| **Cognitive domain** | **Core cognitive tests** | **Additional cognitive tests** |
| --- | --- | --- |
| ***Global cognitive functioning*** | MoCA^1^ | MMSE |
| ***Attention and processing speed*** | D-KEFS Stroop test:   - *time to perform D1* - *time to perform D2*   WAIS-III or IV Coding.  Trail Making Test: *time to perform A.* | CPT :   - Omission errors - Variability in reaction time   WAIS-III Digit span forward: *longest sequence.*  Bell’s test: *omissions.* |
| ***Executive functioning*** | Trail Making Test: *time to perform B-A.*  D-KEFS Stroop test:   - *time to perform D3-1* - *time to perform D4-3* | Tower of London   - *number of movements* - *total time*   CPT: commission errors  WAIS-III Digit span backwards: *longest sequence.* |
| ***Learning and memory*** | Rey Auditory Verbal Learning Test:   - *Sum of the 5 free recalls* - *Immediate recall* - *Delayed recall*   Logical Memory Stories (WMS-III or Rouleau version):   - *Immediate recall* - *Delayed recall* | Brief Visual Memory Test Revised:   - *Immediate recall* - *Delayed recall*   Rey Complex Figure Test:   - *Immediate recall* - *Delayed recall* |
| ***Language*** | Boston Naming Test (30 items version).  Animal fluency. | WAIS-III Vocabulary.  Verbal fluency (letter). |
| ***Visuo-spatial abilities*** | Copy of the Rey Figure | Benton Judgement of Line Orientation.  WAIS-III Blocks design |

Summary of neuropsychological tests performed in all cohorts (i.e., core cognitive tests) and available only in subsamples (i.e., additional cognitive tests). MCI diagnosis was established using core cognitive tests and all available additional cognitive tests.

^1^ Missing data in 3 participants (1 cognitively unimpaired, 2 aMCI).

Abbreviations: aMCI, amnestic Mild Cognitive Impairment; CPT, Continuous Performance Task; D-KEFS, Delis-Kaplan Executive Function System; MMSE, Mini Mental State Examination; MoCA, Montreal Cognitive Assessment; T, time; WAIS, Wechsler Adult Intelligence Scale; WMS, Wechsler Memory Scale.

**Supplementary Table 2: Multiple linear regressions between REM sleep duration and BF volume.**

| **Dependent variable** | **Independent variables** | **Unstandardized coefficient (95% CI)** | **Standard Error** | **Standardized coefficient** | **P_unc._** | **P_FDR-corrected_** |
| --- | --- | --- | --- | --- | --- | --- |
| **Full sample** | | | | | | |
| Ch123 volume | REM-S duration | 0.11 (-0.06 – 0.28) | 0.08 | 0.14 | 0.20 |  |
| Ch4 volume | REM-S duration | 6.95e^-4^ (1.3e^-4^ – 0.001) | 2.8e^-4^ | 0.30 | **0.017** | **0.026** |
| Total BF volume | REM-S duration | 0.54 (0.1 – 0.98) | 0.22 | 0.28 | **0.016** | **0.026** |
| **Cognitively unimpaired participants** | | | | | | |
| Ch123 volume | REM-S duration | 0.04 (-0.15 – 0.23) | 0.09 | 0.06 | 0.68 |  |
| Ch4 volume | REM-S duration | 9.8e^-5^ (-4.9e^-4^ – 6.8e^-4^) | 2.8e^-4^ | 0.06 | 0.73 |  |
| Total BF volume | REM-S duration | 0.11 (-0.35 – 0.56) | 0.22 | 0.07 | 0.63 |  |
| **aMCI participants** | | | | | | |
| Ch123 volume | REM-S duration | 0.22 (-0.09 – 0.52) | 0.15 | 0.25 | 0.17 |  |
| Ch4 volume | REM-S duration | 0.01 (2.3e^-4^ – 0.002) | 5.1e^-4^ | 0.45 | **0.019** | **0.03** |
| Total BF volume | REM-S duration | 1.03 (0.22 – 1.84) | 0.4 | 0.44 | **0.015** | **0.03** |

Results of multiple regressions performed in the full sample and subgroups stratified by cognitive status, between REM sleep duration (reported in minutes) and the volume of BF subregions (i.e., total BF, Ch1-2-3 and Ch4), controlling for age, sex and the total intracranial volume. Results indicated in bold survived an FDR correction for multiple comparisons.

Abbreviations: aMCI, amnestic mild cognitive impairment; BF, basal forebrain; CI, confidence interval; FDR, false discovery rate; REM-S, rapid eye movement sleep.

**Supplementary Table 3: Multiple linear regressions between NREM sleep and wakefulness variables and BF volume.**

| **Dependent variable** | **Independent variables** | **Unstandardized coefficient (95% CI)** | **Standard Error** | **Standardized coefficient** | **P_unc._** |
| --- | --- | --- | --- | --- | --- |
| Ch123 volume | Wakefulness (%) | -0.14 (-0.49 – 0.21) | 0.17 | -0.09 | 0.43 |
|  | N1 sleep (%) | -0.05 (-0.53 – 0.43) | 0.24 | -0.02 | 0.84 |
|  | N2 sleep (%) | 0.11 (-0.34 – 0.56) | 0.22 | 0.06 | 0.62 |
|  | N3 sleep (%) | -0.16 (-0.63 – 0.32) | 0.24 | -0.08 | 0.52 |
| Ch4 volume | Wakefulness (%) | 4.8e^-4^ (-7.3e^-4^ – 0.002) | 6.05e^-4^ | 0.10 | 0.43 |
|  | N1 sleep (%) | -8.3e^-4^ (-0.002 – 8.4e^-4^) | 8.3e^-4^ | -0.14 | 0.32 |
|  | N2 sleep (%) | 5.6e^-4^ (-0.001 – 0.002) | 7.8e^-4^ | 0.10 | 0.48 |
|  | N3 sleep (%) | -0.001 (-0.003 – 4.9e^-4^) | 8.2^e^-4 | -0.19 | 0.17 |
| Total BF volume | Wakefulness (%) | 0.14 (-0.81 – 1.09) | 0.47 | 0.04 | 0.77 |
|  | N1 sleep (%) | -0.56 (-1.86 – 0.74) | 0.65 | -0.11 | 0.39 |
|  | N2 sleep (%) | 0.46 (-0.75 – 1.67) | 0.61 | 0.09 | 0.45 |
|  | N3 sleep (%) | -0.89 (-2.16 – 0.38) | 0.64 | -0.18 | 0.17 |

Results of multiple regressions performed with NREM sleep and wakefulness duration or proportion as predictors, and the volume of BF subregions (i.e., total BF, Ch1-2-3 and Ch4) as outcomes, separately, controlling for age, sex and the total intracranial volume.

Abbreviations: BF, basal forebrain; CI, confidence interval; NREM-S, non-rapid eye movement sleep.

**Supplementary Table 4: Partial correlations between REM sleep duration and the volume of control brain regions.**

| **Variable** | | **REM sleep duration (%)** |
| --- | --- | --- |
| **Full sample (n=62)** |  |  |
| Total GMV (cm^3^) | r (95% CI) | -0.08 (-0.30 – 0.13) |
|  | p-value | 0.54 |
| ACC volume (mm^3^) | r (95% CI) | 0.05 (-0.18 – 0.26) |
|  | p-value | 0.72 |
| Amygdala volume (mm^3^) | r (95% CI) | 0.14 (-0.11 – 0.34) |
|  | p-value | 0.28 |
| Cuneus volume (mm^3^) | r (95% CI) | -0.08 (-0.35 – 0.19) |
|  | p-value | 0.55 |
| Hippocampus volume (mm^3^) | r (95% CI) | 0.16 (-0.10 – 0.36) |
|  | p-value | 0.22 |
| ITG volume (mm^3^) | r (95% CI) | -0.07 (-0.30 – 0.14) |
|  | p-value | 0.59 |
| PCC volume (mm^3^) | r (95% CI) | -0.12 (-0.37 – 0.15) |
|  | p-value | 0.37 |
| Precuneus volume (mm^3^) | r (95% CI) | -0.20 (-0.40 – 0.06) |
|  | p-value | 0.14 |
| SFG volume (mm^3^) | r (95% CI) | -0.09 (-0.34 – 0.14) |
|  | p-value | 0.48 |
| SMG volume (mm^3^) | r (95% CI) | 0.13 (-0.15 – 0.38) |
|  | p-value | 0.33 |
| **MCI group (n=31)** |  |  |
| Total GMV (cm^3^) | r (95% CI) | -0.01 (-0.38 – 0.35) |
|  | p-value | 0.97 |
| ACC volume (mm^3^) | r (95% CI) | 0.04 (-0.36 – 0.40) |
|  | p-value | 0.86 |
| Amygdala volume (mm^3^) | r (95% CI) | 0.20 (-0.13 – 0.47) |
|  | p-value | 0.31 |
| Cuneus volume (mm^3^) | r (95% CI) | -0.18 (-0.55 – 0.30) |
|  | p-value | 0.36 |
| Hippocampus volume (mm^3^) | r (95% CI) | 0.26 (-0.15 – 0.55) |
|  | p-value | 0.18 |
| ITG volume (mm^3^) | r (95% CI) | 0.04 (-0.34 – 0.35) |
|  | p-value | 0.85 |
| PCC volume (mm^3^) | r (95% CI) | 0.22 (-0.20 – 0.59) |
|  | p-value | 0.25 |
| Precuneus volume (mm^3^) | r (95% CI) | -0.13 (-0.53 – 0.32) |
|  | p-value | 0.52 |
| SFG volume (mm^3^) | r (95% CI) | 0.02 (-0.39 – 0.47) |
|  | p-value | 0.92 |
| SMG volume (mm^3^) | r (95% CI) | 0.09 (-0.32 – 0.53) |
|  | p-value | 0.65 |

Table showing the result of partial correlations between the volume of control regions and REM sleep percentage, controlling for age, sex and the TIV. Results in bold are significant at the p<0.05 (uncorrected) level. Confidence intervals are based on 1000 bootstrap replicates.

Abbreviations: ACC, anterior cingulate cortex; BF, basal forebrain; Ch4, nucleus basalis of Meynert; GMV, gray matter volume; ITG, inferior temporal gyrus; PCC, posterior cingulate cortex; REM, rapid eye movement; SFG, superior frontal gyrus; SMG, supramarginal gyrus.
